# Precision oncology: as much expectations as limitations

**DOI:** 10.3332/ecancer.2018.ed86

**Published:** 2018-11-29

**Authors:** Vitor Fiorin Vasconcellos, Leandro Machado Colli, Ahmad Awada, Gilberto de Castro Junior

**Affiliations:** 1 Clinical Oncology, Instituto do Câncer do Estado de São Paulo, Hospital das Clínicas da Faculdade de Medicina da Universidade de São Paulo, São Paulo, SP, Brazil; 2 Division of Cancer Epidemiology and Genetics, National Cancer Institute, National Institutes of Health, Bethesda, MD, USA; 3 Medical Oncology, Institut Jules Bordet, Université Libre de Bruxelles, Brussels, Belgium

**Keywords:** precision medicine, molecular targeted therapy, sequence analysis, DNA, molecular diagnostic techniques

## Abstract

It is encouraging to witness the recent price reduction and expanded access to next generation sequencing platforms, the increasing number of investments and publications on new targets and respective targeted drugs, as well as the worldwide excitement with anti-cancer personalised therapies. This editorial aims to highlight the limitations regarding the small proportion of solid cancers potentially eligible for the use of molecular-based targeted drugs until now. It also covers the expected clinical benefits in refractory patients treated by matched therapies, and detailed cost-effectiveness analysis of the use of DNA sequencing analysis oncology practice in an academic and large-scale community.

The major goal of personalised cancer therapy is to identify druggable tumour genomic alterations that can improve patients’ quality of life and survival. The feasibility of large-scale clinical use of precision medicine, the impact of actionable matched treatments and the most adequate value-based genomics strategies are not yet well established [1].

The implementation of targeted therapies was a revolutionary landmark in oncology. Significant gains in clinically relevant outcomes have been achieved in several types of cancer such as gastrointestinal stromal tumours (GIST), EGFR-mutated and ALK-fused non-small cell lung cancer, all-RAS wild-type colorectal cancer, BRAF V600 melanoma, ovarian BRCA-deficient, HER-2 breast and gastroesophageal cancers, agnostic high microsatellite instability solid tumours [2] and TRK-fused tumours [3].

However, targeted therapy development requires several years of intense multidisciplinary effort, from understanding the cancer biology to the goal of testing a new drug during a phase III study. As a result, it is important to recognise that at this point the magnitude of predictive biomarkers is restricted to certain subtypes of solid tumours. Even in countries where international regulatory agencies have more liberal incorporation policies, the coverage of next-generation sequencing (NGS) molecular testing and personalised treatments is limited. For example, it is estimated that for all US patients diagnosed with advanced or metastatic cancer in 2018, only 8.33% would be eligible for genome-driven drugs and only 4.9% would benefit from using personalised therapies [2]. Basket trials are one opportunity for expanding the number of solid tumours and subtypes with predictive biomarkers. The implementation of the anticancer targeted agents to match a molecular abnormality remains a major limitation to precision oncology.

Two meta-analyses with 346 phase I and 560 phase II studies suggested statistically significant gains in progression-free survival (PFS) and overall survival (OS), respectively, when biomarker based treatment strategies were used [4, 5]. In contrast, a randomised phase II SHIVA trial did not show benefit in PFS, response rate or even less toxicity with targeted therapies based on somatic mutations in comparison to investigators choice of treatment [6], dissuading targeted therapy off-label use.

Interpretation and validation of precision oncology studies are especially challenging because of their inherent pre- and post-analytical complexity: epidemiological and genetic variability between populations, individual tumour heterogeneities, sample process requirements, NGS sequencers techniques, genomic panels covered, and bioinformatics analysis differ widely between the trials and commercially available platforms. In this context, we believe that a multidisciplinary molecular tumour board integrative case discussion should be standard practice in order to minimise pitfalls, maximise holistic data interpretations and optimise matched-therapy guided orientation [7]. A better understanding of cancer biology can be another advantage of discussing these patients in the molecular tumour boards.

Last but not least, we must consider the cost effectiveness of the required infrastructure necessary to enable routine practice of precision cancer medicine from the academic, community and individual perspectives. Among community treated advanced NSCLC, broad-based genomic sequencing–tested patients did not benefit from improved survival in comparison to routine EGFR- and ALK-tested patients [8]. In an individual-based intervention study by the public health services of Norway, which included cost modelling, a single-agent biomarker based approach was 2.5 times more expensive than best supportive care (BSC) [9]. Moreover, in refractory metastatic lung adenocarcinoma, the NGS target therapy produced a gain of only 0.009 QALYs, resulting in an Incremental Cost-Effectiveness Ratio (ICER) of USD 363,078/QALY in comparison to chemotherapy or BSC [10].

## Conclusion

In conclusion, it is encouraging to witness the recent price reduction and expanded access to NGS platforms, increasing number of publications on new targets and respective targeted drugs, investments by the pharmaceutical industry and excitement within the oncology community over personalised therapies. Our greatest challenge, however, continues to be adequate patient selection, development and access to clinical trials, tumour board decision process, incorporation of cost effectiveness analysis and being honest about patients’ and family members’ expectations and frustrations regarding the heterogeneity of our precision. Precision oncology is in the earliest stages of development and in expanding our knowledge in this field we look forward to a time when our expectations surpass current limitations.

## Conflicts of interest

We declare that we have no conflicts of interest.
